# Relationship of 2D Affinity to T Cell Functional Outcomes

**DOI:** 10.3390/ijms21217969

**Published:** 2020-10-27

**Authors:** Elizabeth M. Kolawole, Tracey J. Lamb, Brian D. Evavold

**Affiliations:** Department of Pathology, University of Utah, 15 N Medical Drive, Salt Lake City, UT 84112, USA; emkolawole@path.utah.edu (E.M.K.); tracey.lamb@path.utah.edu (T.J.L.)

**Keywords:** pMHC, TCR, affinity, bond lifetime, T cells, 2D kinetics

## Abstract

T cells are critical for a functioning adaptive immune response and a strong correlation exists between T cell responses and T cell receptor (TCR): peptide-loaded MHC (pMHC) binding. Studies that utilize pMHC tetramer, multimers, and assays of three-dimensional (3D) affinity have provided advancements in our understanding of T cell responses across different diseases. However, these technologies focus on higher affinity and avidity T cells while missing the lower affinity responders. Lower affinity TCRs in expanded polyclonal populations almost always constitute a significant proportion of the response with cells mediating different effector functions associated with variation in the proportion of high and low affinity T cells. Since lower affinity T cells expand and are functional, a fully inclusive view of T cell responses is required to accurately interpret the role of affinity for adaptive T cell immunity. For example, low affinity T cells are capable of inducing autoimmune disease and T cells with an intermediate affinity have been shown to exhibit an optimal anti-tumor response. Here, we focus on how affinity of the TCR may relate to T cell phenotype and provide examples where 2D affinity influences functional outcomes.

## 1. Introduction

Recognition of pMHC by TCR is the initial trigger for activation of T cells and provides a gateway to the adaptive immune response. The affinity of TCR:pMHC interactions is an important initial regulatory parameter in activation. Although the distal role of T cells in the outcome of the immune response is widely investigated, fewer studies focus on the most proximal, engagement of TCR with pMHC. The bond between pMHC and TCR is often described as being relatively weak [1,2] in terms of 3D affinity and compared to antibodies [3,4,5], yet TCR:pMHC interactions are highly sensitive [6] with T cells responding to as few as 1–10 pMHCs on an antigen presenting cell [7,8,9]. This interaction is also decidedly specific [10] with the ability to differentiate single amino acid substitutions in peptide sequences that are then translated into differential phenotypic responses [11]. This sensitivity, specificity, and the extent to which a naïve T cell receives stimulation via the TCR contributes to the activation status and phenotype of that cell. In this review, we discuss the emerging body of work illustrating the significance of TCR two-dimensional affinity for pMHC (which is not to be confused with TCR avidity for pMHC) in the initiation and dissemination of T cell signaling during attack of invading pathogens and autoimmune conditions.

## 2. The Affinity Measurements of TCR

The initial development of the T cell repertoire in the thymus results from the combination of both positive and negative selection to create a T cell population that is MHC restricted [12,13,14]. In a polyclonal response any one epitope can be recognized by T cells with differing affinities. The affinity of TCR for any particular pMHC can be measured in a number of different ways.

### 2.1. 3D Methods of Measuring Affinity

**Figure 1:**
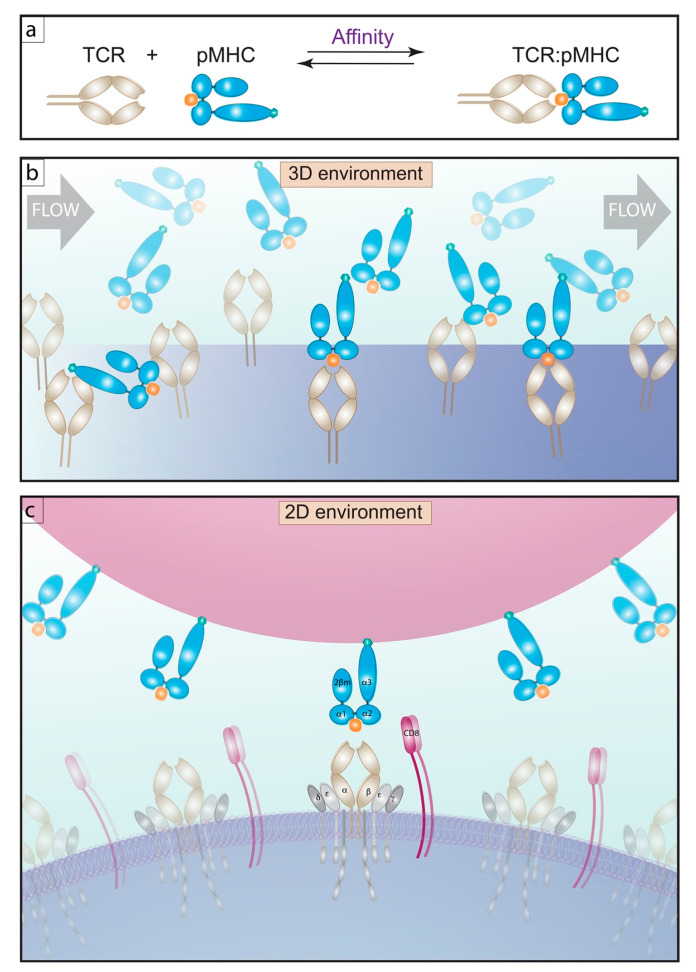
T cell affinity for pMHC, 3D versus a 2D environment. (**a**) schematic of an affinity reaction. (**b**) 3D affinity measurements by SPR, demonstrates soluble pMHC flowing over TCR bound to a sensor. Beneath this surface a detector records the change of resonance angle, giving a read out of affinity as the number of molecules per volume, μM^−1^. (**c**) 2D affinity measurements by 2D-MP show the interaction of TCR embedded in live T cells interacting with pMHC bound to surrogate APC’s in the form of red blood cells (RBCs), these two cells are mechanically brought into contact and adhesion frequency is measured. The 2D affinity is read out as the number of molecules per area, μm^4^.

Surface plasmon resonance (SPR) provides a method for determining receptor-ligand affinity of purified proteins in a three-dimensional (3D) environment, whereby the purified mobile pMHC flows across purified immobilized TCR (Figure 1) yeilding an affinity measurement between TCR and cognate pMHC in the range of 1–100 μM [15,16,17,18,19,20,21,22,23,24,25,26,27,28,29,30]. This system, while deriving TCR:pMHC affinity for defined antigens does not always capture the full dynamic range of antigen specific T cells missing responses dominated by lower affinity TCRs that occur in autoimmune disease and the immune response to cancer [23]. Furthermore, these types of analyses, while carried out using purified proteins (Figure 1a), also lack any localized influence provided by the complex membrane environment. In the case of membrane embedded proteins their interactions are constrained to fewer degrees of freedom and are governed by two dimensional (2D) parameters. Therefore, SPR allows for the analysis of receptor-ligand affinity in a reductionist format with purified proteins at the cost of accounting for the native cellular membrane milieu and any influence bestowed on TCR:pMHC interactions. A correlation between 3D affinity and T cell function can exist [31,32,33,34], but the 3D measurements do not changes in peptide [17,35,36,37]. Furthermore, SPR requires production of multiple purified TCRs to fully capture the breath of affinities present during a polyclonal immune response. Thus, measurements of TCR affinity in 3D may differ from biological responses [16,17,18,19,24,28,30,35,38,39].

### 2.2. 2D Methods of Measuring Affinity

2D-based affinity measurements such as fluorescence based methods fluorescence recovery after photobleaching (FRAP) [40,41] and fluorescence resonance energy transfer (FRET) [42] (which are discussed elsewhere) [43,44] and the 2D micropipette adhesion frequency assay (2D-MP) [35,45,46,47] (Figure 1c) have advantages to measure both receptor and ligand as embedded within membranes. This allows for the investigation into how alteration of TCR affinity for pMHC operates in the context of the organization of the plasma membrane complete with CD3 proteins, co-receptor, Lck, lipid raft, actin cytoskeleton, and other proteins [35,42,48]. By nature, 3D affinity cannot fully replicate the biology of this interaction. This is perhaps best exemplified in the OT-I receptor transgenic altered peptide ligand (APL) system. Huang et al. analyzed six OT-I APLs which elicit varying degrees of immune activation and found direct correlates between varying 2D affinity (µm^4^) and on-rates (µm^4^ s^−1^). In contrast, the 3D affinity (uM^−1^) and 3D on-rate (uM^−1^s^−1^) did not equate to response magnitude [35]. Furthermore, 2D kinetic rates were more sensitive than their 3D counterparts. This represented a 1000-fold change for 2D affinity and an almost 10,000-fold change in 2D on-rate compared with a ~15-fold change in 3D affinity and <10-fold change in 3D on-rate. In addition, 2D assays can measure affinity for ligands such as antagonists and self-epitopes involved in positive selection of thymocytes that are not easily detected by tetramer and 3D-based assays. Therefore, the increased sensitivity of 2D as compared to 3D is a clear advantage to 2D assays.

## 3. Are Lower Affinity T Cells Real?

### 3.1. Low Sensitivity Techniques

Much of what we know about the frequency and response of antigen reactive T cells is limited by the methods of detection. Enumeration of antigen-reactive T cells through the use of pMHCI and pMHCII tetramers and multimers (oligomers) has become the standard procedure due to increased reagent availability and convenience of flow cytometry-based analysis [49,50,51,52]. Although tetramer staining does not always reflect 3D affinity measurements, particularly in tumor and autoimmune models, the use of these highly valued techniques has shown a correlation with the number of cells identified by tetramer and cytokine production [23,53]. This begs the question as to the importance of the lower affinity T cells identified by 2D analyses.

Upon examination of differences in how these two different technologies measure T cell reactivity, it can be justified that lower affinity T cells will not be detected by tetramer and oligomer technologies for the following reasons. Firstly, tetramer technology is based on enhancing the TCR avidity by providing multivalent interactions [52,54,55,56,57,58,59,60]. The chemical definition of affinity is the equilibrium constant between on and off rates. Affinity in the context of immunological interactions can be described as the likelihood of bond formation between a single TCR:pMHC pairing as high affinity T cells have a high probability of bond formation compared to low affinity T cells, which have a low probability of forming bonds and propagating TCR signaling. Tetramers, however, measure the avidity of several bonds between multiple recombinant pMHC molecules. The monomeric binding of the first arm of pMHC in a tetramer to TCR increases stability of the complex and alters the kinetic rates to allow successive pMHC arms of the tetramer to bind [61]. This avidity interaction requires a minimum affinity for the first monomer to bind that will thereby underestimate the number of antigen reactivity cells [16,62]. Secondly, it is often assumed incorrectly that the intensity of tetramer staining equates with TCR affinity. While tetramer can identify the higher affinity T cells, often the intensity does not equate to TCR affinity [16,61,63,64,65,66]. Thirdly, TCR density can affect tetramer interaction [55,67]. This means that T cells that express a low density of TCRs would fail to stain with tetramers even if the TCR affinity is high. Lastly, it is difficult to simultaneously measure tetramer and intracellular cytokines by flow cytometry in response to antigen due to internalization of the TCR upon stimulation. This makes it challenging to attribute cytokine production solely to tetramer positive cells.

### 3.2. Specificity with High Sensitivity by 2D-Micropipette

**Figure 2:**
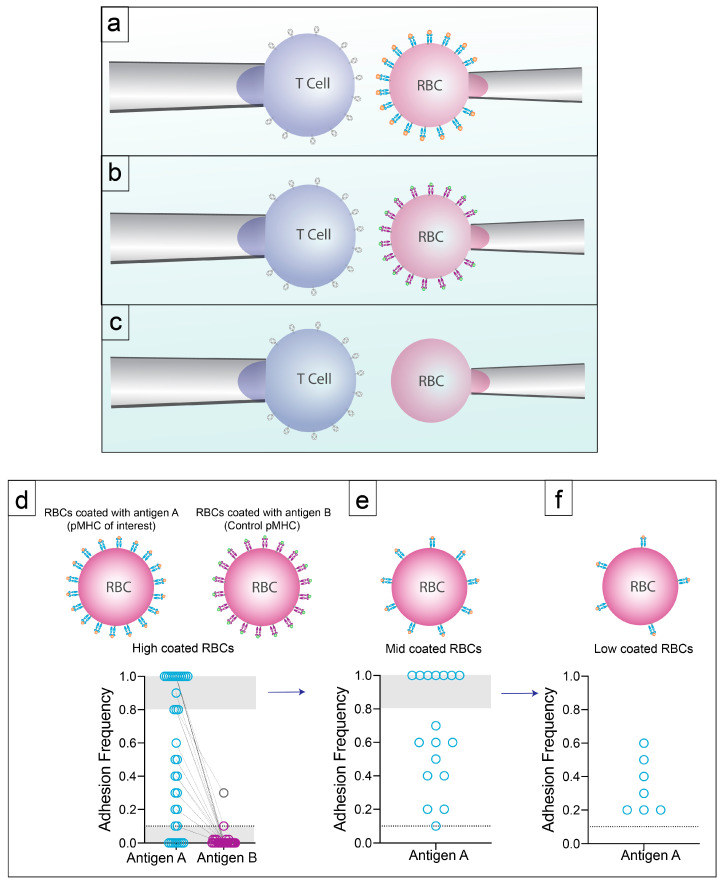
Controls and Specificity. (**a**) T cells of interested are aspirated onto a pipette with high density pMHC-coated hRBCs aspirated onto an opposing pipette. Cells are brought into contact using a piezoelectric actuator 50 times. (**b**) If binding is detected with pMHC of interest between the range of 10% and 80% the hRBC is switched out for a hRBC coated with an irrelevant pMHC to test specificity. (**c**) T cells tested to hRBCs without pMHC to test for non-specific binding between the T cell and non pMHC molecules on the hRBC. (**d**) Adhesion frequency of each cell tested to pMHC of interest and irrelevant pMHC. (**e**) If the pMHC of interest has an adhesion frequency of >80% the hRBC is replaced with a hRBC coated with a lower pMHC density. (**f**) If using a lower pMHC density still yields an adhesion frequency of >80% we continue to move to a lower pMHC density. Following this, 2D affinity can be calculated.

The main discrepancy between tetramers and the frequency of antigen reactive T cells by 2D-based techniques lies in the sensitivity of these respective assays. Related to sensitivity is the requirement for specificity. In the case of tetramers, non-specific tetramers are often used to provide the background reactivity in the sample, although it seems this control is sometimes overlooked. Similarly, the 2D-micropipette measurements are routinely compared to other pMHC monomers as a control for specificity. In a polyclonal immune response, the 2D-micropipette has the sensitivity to derive affinity for single receptor-ligand interactions with affinities ranging anywhere from 10^−1^ to 10^−7^ µm^4^. This sensitivity will detect T cells expressing lower amounts of TCR that may be missed by other methods, such as tetramer staining. For each model system and each set of experiments, T cells are tested to a range of pMHC-coated red blood cells (RBCs) concentrations. Those RBCs with the highest concentration of pMHC are able to detect the lowest affinity T cells and those with the lowest concentration are able to detect the highest affinity T cells. A T cell of interest is first aspirated onto a pipette with a pMHC-coated human RBC (hRBC) possessing a high density of monomer on an opposing pipette (Figure 2a). When binding occurs between the two opposing cells an adhesion frequency is calculated [68]. T cells are additionally probed with RBCs coated with antigen-irrelevant pMHC monomers (Figure 2b) and those devoid of pMHC (Figure 2c) to determine any rates of non-specific binding [63,69,70,71]. In the case of CD4+ T cells this control is often Class II invariant chain peptide (CLIP) as it is provided by the NIH tetramer core facility as the control for MHC class II tetramers due to the property that it binds to most MHC class II alleles. If binding with the high concentration of pMHC is >80% the T cell is tested to a lower pMHC concentrations (Figure 2d–f) until a binding frequency of <80% is reached. Use of an adhesion frequency range of 10–80% allows for exclusion of the extreme limits of detection on any given RBC [35,69]. Adhesion frequency in conjunction with the density of TCR on the T cell and pMHC on the RBC allow for the calculation of 2D affinity (Box 1). Utilization of these controls has demonstrated that low affinity T cells, similar to higher affinity TCRs possess specificity to distinguish between the antigen-loaded pMHC of interest and control pMHC.

Box 1 **Defining TCR affinity for pMHC.**

In immunology, affinity is the probability of bond formation that can often times be a predictor of cell function or signal strength particularly when used in conjunction with bond lifetime. The affinity of TCR for pMHC (association constant *K_a_*) is derived from the on/off rates (*K_on_*) and (*K_off_*) of this interaction at equilibrium [16,72]. The effective 2D affinity is encompassed by the equation *A_c_K_a_* = −ln(1−*P*_a_) *m*_r_ *m*_l_, where the affinity (*K_a_)* and contact area (*A_c_*) between the T cell and surrogate APC (pMHC coated RBC) require quantification of the receptor density (*m*_r_), the ligand density (*m*_l_), and the frequency of adhesion (*P*_a_) to be calculated.

Adhesion frequency (*P*_a_): When the two cells are brought into contact for an equilibrium contact time of two seconds, deformation and stretching of the highly flexibly RBC membrane upon separation of the two cells denotes an adhesion event and is recorded as a binding event and given a score of 1. Lack of observed stretching denotes a non-binding event and is recorded as a score of 0 [68]. These set contact and retraction cycles (at equilibrium) are controlled by an electric piezo actuator for 50 contacts.

TCR receptor density (*m*_r_) and pMHC RBC density (*m*_l_): T cells and pMHC coated RBCs are stained with anti-TCRβ PE and anti-MHC class II PE antibodies, respectively, and run by flow cytometry. Densities of pMHC and TCR are calculated using BD QuantiBrite Beads. Molecules per area were calculated by dividing the number of TCR and pMHC per cell by the respective surface areas (hRBC 140 mm^2^, T cell during assay measured diameter of an individual T cell and the surface area equation of a sphere [35]).This measurement is of a single TCR-pMHC molecular interaction has long been expected as a predictor of T cell responses but this is not always the case [73]. Despite this definition of the reactants as purified proteins (Figure 1a), the affinity for T cells is more complex and often somewhat misapplied in immunology, especially to the TCR and its interaction with pMHC. To bypass some of the early difficulties in producing the purified proteins necessary to measure these interactions, “affinity” was often inferred indirectly by comparing functional readouts of different TCRs to different clones of the same antigen or through the use of altered peptide ligands (APLs) with different levels of functional potency that assumed different affinities for a single TCR clone [37,74,75,76]. The advent of pMHC tetramer technology also allowed a relatively easy measure of avidity to TCRs to pMHC by flow cytometry. While the highest affinity T cells are incorporated with tetramer staining, affinity is not an avidity interaction, nor is it the direct functional response often called strength of signal.

## 4. Expansion and Frequency of Low Affinity CD4+ T Cells

This specificity of low and high affinity TCRs, coupled with the sensitivity of the 2D-micropipette assay, provides a mechanism to define the frequency of antigen-reactive T cells directly ex vivo or following in vitro culture across the entire range of responding cells. For situations where higher affinity antigen-TCR:pMHC interactions are more prominent, tetramer and 2D-micropipette provide different views of the T cell response with tetramers underestimating the T cell response. One example during infection is the expanded mixed affinity profile of low and high affinity CD4+ T cells that respond to the dominant MHC-II epitope glycoprotein (GP)_66–77_ of lymphocytic choriomeningitis virus (LCMV). At the peak of the immune response to LCMV, ~10% of splenic CD4+ T cells react to GP_66–77_ by tetramer staining compared to ~45% by 2D-micropipette [69]. Self-reactive T cells often express TCRs of low affinity with most harboring TCRs with affinity too low to bind to tetramers [49,51,55,62,67,77,78,79,80]. Thus, when 2D-micropipette is used to look at antigen-reactivity of CD4+ T cells in autoimmune diseases such as the Experimental Autoimmune Encephalomyelitis (EAE) mouse model of Multiple Sclerosis (MS), the frequency of autoreactive CD4+ T cells is much higher than previously appreciated using tetramer staining. At peak disease, 2D micropipette detected ~80% MOG_38–49_ reactive CD4+ T cells in the CNS, ~70% in the draining cervical lymph nodes and ~15% in the spleen [81] compared to 4–8% in the CNS, and < 1% for both draining cervical lymph nodes and the spleen that was stained with MOG_38–49_ tetramer. Similarly, in diabetes analysis of the frequency of hybrid insulin peptide (HIP2.5)-reactive T cells by micropipette detected ~40% antigen reactivity in pancreatic islets of diabetic NOD mice compared to ~4% by tetramer [71]. Also for the diabetogenic insulin antigen (InsB_9–23_), the frequency of CD4+ T cells was below the limit of detection for tetramer, yet 2D-micropipette revealed 58% and 47% InsB_9–23_-reactive CD4+ T cells in the islets and spleen, respectively [82]. Use of 2D-micropipette to measure antigen-reactive CD4+ T cells has thus begun to alter our understanding of disease progression during infection and autoimmunity by demonstrating that there is a much higher frequency of antigen specific CD4+ T cells than previously appreciated.

Polyclonal 2D affinity is a functionally relevant metric. The low precursor frequency of antigen-reactive T cells to any antigen dictates that all antigen-reactive cells identified, regardless of affinity, were detected in the assay because they underwent cell division and expanded in number. This is illustrated in the situation of T1D where HIP-reactive CD4+T cells are predominantly measured in the organ of relevance (the islets) but not in the spleen where their frequency is rare among all of the other T cell specificities [71]. Precursor frequency of quiescent peripheral T cells with lower or high affinity TCRs has been shown to influence the profile of activated T cells [83]. For example, naïve precursor frequencies of high affinity T cells identified by tetramers correlated with immunodominance of foreign antigen-specific CD4+ T cells as measured by tetramer following immunization. Of interest, enumeration of naïve precursor frequency and expanded T cells as measured by 2D-micropiette yields a more robust correlation owing to the inclusion of lower affinity antigen-reactive T cells that evade tetramer detection [84]. The 2D-micropipette data is supported by in vivo limiting dilution where the majority of antigen-reactive clones were low affinity and tetramer negative across several CD4+ T cell responses specific for influenza, LCMV, mycobacterium, flagellin, and myelin specific antigens demonstrating this broadly applicable principle [84].

## 5. The Affinity of TCR for pMHC Modulates TCR-Derived Signals

Data from the 2D-micropipette assay in a variety of systems have revealed that lower affinity cells not only expand, but can also dominate polyclonal T cell expansion in the majority of systems tested [63,69,70,81,82,84,85,86,87]. Thus, it is pertinent to consider the functional ramifications for a T cell that becomes activated via a lower affinity TCR. Both lower affinity and higher affinity T cells can become activated to similar levels. This has been demonstrated in vitro by analysis of cultured polyclonal MOG_35–55_-reactive or LCMV-reactive CD4+ T cells pulsed with MOG_35–55_ or GP_61–80_, respectively. After 1 week in culture both tetramer positive (higher affinity) and tetramer negative (lower affinity) produce similar levels of cytokines [63]. It has also been repeatedly shown that lower affinity T cells are capable of the host of effector functions needed for CD4+ T cell mediated autoimmune disease [63,81].

Although possible differences in effector function between higher and lower affinity T cells may exist, this analysis is currently ongoing. What are the differences in the molecular interactions that govern the activation of T cells in lower affinity T cells compared to higher affinity TCRs? It is possible that T cells with lower affinity TCRs simply have less of a chance of becoming activated due to potentially less contact time with a pMHC-displaying APC. Signaling from the TCR complex occurs in both higher and lower affinity T cells as evidenced by calcium flux [88,89] and zeta chain phosphorylation [90], albeit to potentially different levels. The early biochemical and kinetic parameters mediated by TCR:pMHC governs the extent of signal propagation. Thus, with high on-off rates on lower affinity T cells, TCRs may collectively have less time to propagate signals that emanate from both the TCR signaling complex and potentially through other molecules that are involved in T cell activation such as co-stimulatory molecules. Variation in signal strength can modulate the transcriptional pathways that are activated and potentially could lead to different functional properties. For example, self-reactive human CD4+ T cells expressing lower 2D affinity TCRs that do not stain well with tetramers formed unusual immunological synapses [86]. This may indicate a mechanism by which low accumulation of pMHC and TCR at the immune synapse of lower affinity T cells may influence function.

## 6. Factors Influencing Expansion of Lower Affinity T Cells in Addition to 2D Affinity of the TCR for pMHC

Although detection of lower affinity CD4+ T cells occurs after expansion, a currently unknown factor is whether the affinity profile of T cells expanded from the precursor pool may be influenced by the type of antigen presenting cell (APC). It is possible that different APC subsets may influence the interaction with TCRs via differences in pMHC density and the amount and/or types of surface molecules that provide co-stimulatory signals. This could influence the breadth in the affinity of the responding T cells over and above TCR specificity by altering the strength of TCR signaling and TCR:pMHC bond lifetimes that emanate from high and low affinity TCRs upon ligation with pMHC.

### 6.1. Differential Signaling from Co-Receptors

**Figure 3:**
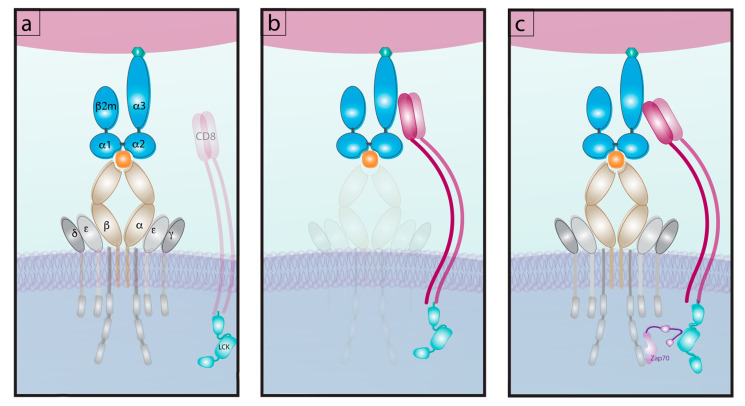
The interactions of pMHC with TCR and co-receptor. (**a**) TCR affinity interaction for pMHC excluding CD8 co-receptor. (**b**) CD8 Affinity interaction for pMHC excluding TCR. (**c**) The tri molecular interaction of TCR, CD8 and pMHC read out as “normalized adhesion bonds” and the engagement of Lck with Zap70.

While many studies focus on the interaction of TCR:pMHC (Figure 3a), the CD4 and CD8 co-receptors augment signaling downstream of TCR:pMHC by recruiting Lck to the CD3 signaling complex [91]. This alters the overall strength of binding [54] which could be important for lower affinity T cells by providing compensatory interactions to increase strength of TCR signaling [77]. Both CD8 and CD4 co-receptors have relatively weak affinities for their respective MHC with the affinity of CD8 for MHC-I being higher than that of CD4 for MHC II by both 2D and 3D measures [91,92]. CD8 affinity for MHC as a confounding factor in identifying TCR affinity is controlled for in 2D-micropipette assays via the use of mutated pMHC-I monomers that cannot bind to CD8 (Figure 3a). One can also use intact pMHC and define the affinity of CD4 and CD8 co-receptors for MHC (Figure 3b), as well as probe the interaction of all three molecules termed “normalized adhesion bonds” (Figure 3c) [92,93,94] for a more complete analysis of these three interacting molecules directly ex vivo.

### 6.2. Bond Lifetime under Force: A Key Predictor of T Cell Function

**Figure 4:**
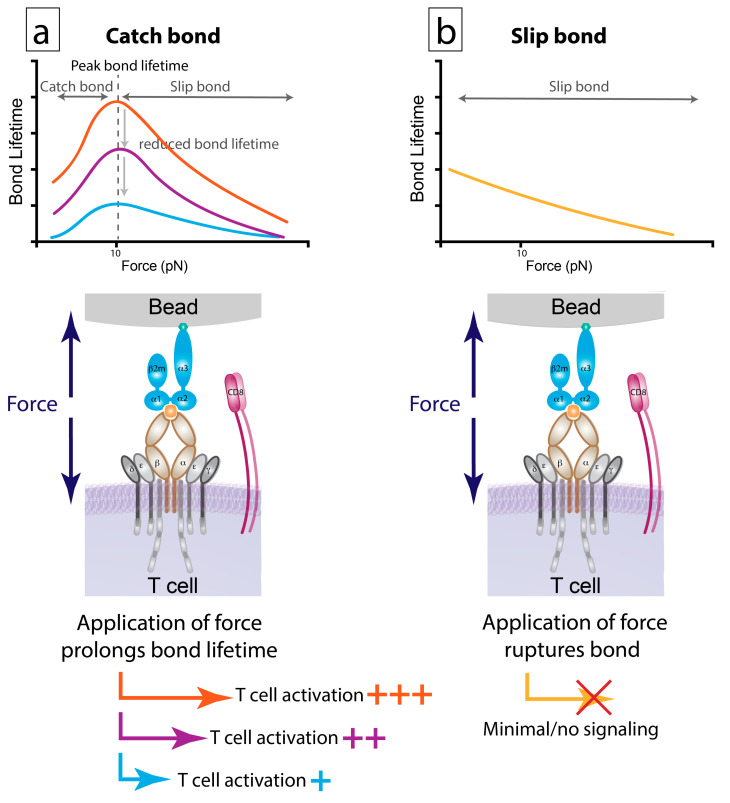
Catch versus slip bonds. (**a**) Catch bonds: As increasing amounts of force are applied to the TCR:pMHC complex bond lifetime increases until a pinnacle is reached. Continuing to increase force at this point results in decreases bond lifetime creating a catch bond. 10pN has been demonstrated to be peak force. Longer peak bond lifetimes result in increases T cell signaling. (**b**) A slip bond: as force is increased, bond lifetime decreases. Slip bonds generate minimal to no force.

Once TCR engages with pMHC, the kinetic on- and off-rates can be influenced by a number of factors [95] that include 2D confinement time [96,97] and bond lifetime under force which can be measured by multiple techniques [98,99,100], which for TCR:pMHC include biomembrane force probe (BFP) [88], atomic force microscopy (AFM) [101,102,103], DNA tension sensor [90] and optical tweezer [104]. T cells apply force to the APC via the TCR:pMHC bond which leads to two types of effects. Catch bonds display increased bond lifetime as force is applied before reaching a peak level. For interactions exhibiting catch bond formation, force has been shown in multiple studies to peak at 10 pico Newtons (pN) for both CD4+ and CD8+ T cells [88,90,93,104,105,106,107,108,109]. Application of additional force after this level decreases the bond lifetime of a catch bond (Figure 4a). In contrast, application of force to a slip bond decreases the bond lifetime from the formation of the bond (Figure 4b). In terms of function, catch bonds are productive bonds giving rise to the longer lived bonds that mediate sustained calcium signaling and increased CD45 exclusion from TCR synapses, allowing TCR signaling to proceed [88,107,109,110]. The magnitude of bond lifetime has been shown to correlate with calcium release and the production of multipotent cytokine producing T cells, a hallmark of efficacious cytotoxic CD8+ T cells during infection [88,93].

Few studies have been carried on bond lifetimes under force since the measurements are currently limited to monoclonal systems due to the labor-intensive analysis of the single molecule interactions. However, some evidence indicates that bond lifetime may supersede the affinity measurements with respect to mediating T cell activation [107]. A recent study identified CD4+ and CD8+ T cells with TCRs that were high affinity and tetramer positive but failed to lead to T cell activation [110]. The failure of T cells harboring these high affinity TCRs occurred despite similar 3D affinity, tetramer staining, crystal structures, and T cell:APC dwell times when compared to T cells containing TCRs which did lead to activation. The key difference was that T cells with stimulatory TCRs formed a catch bond and the non-stimulatory TCR displayed slip bond characteristics. Together, these findings demonstrate that additional factors beyond TCR affinity may also impact T cell fate.

## 7. Affinity Profile and CD4+ T Cell Effector Phenotypes

T cells have a breadth of effector functions and phenotypic fates that can be adopted by T cell subsets. During an immune T cell response, a plethora of factors contribute to the transduction of a T cell signal into a biological response. The extent to which T cell effector function and memory are shaped occurs via modulation of signal duration [111], co-stimulation [112], macromolecular orientation [22,113], and the segregation of phosphatases such as CD45 from the T cell:APC synapse [110,114]. This concert of factors modulates acquisition of effector function in phenotypically different T cell subsets [115] including regulatory mechanisms that dampen effector function. Given that the affinity of the TCRs on a T cell influence many of these factors, it follows that the affinity of a TCRs for pMHC on APCs [35] and the mechanical force that accompanies T cell activation [88,116] could influence the development of different T cell phenotypes.

### 7.1. T Effector Cells (Teff)

The affinity profile of Teff is highly varied. It has long been believed that high affinity interactions between TCR:pMHC give rise to T cells with the ability to acquire effector properties and generate memory [1,117] but existing data does not support this belief. In particular, examination of CD4+ Teff in autoimmune disease is the best demonstration of the functional power of T cells harboring lower affinity TCRs. The need to recognize self-antigen to develop and survive positive selection without the immediate threat of autoimmune disease, coupled with the requirement to cross-react with foreign antigen to mount an effective immune response during infection, leads to the generation of a pool of peripheral T cells that by definition can react with self-protein. TCR affinity for foreign and self-antigens is likely a parameter for distinguishing foreign antigens and establishing self-tolerance [74]. Unlike populations of T cells reacting against foreign antigens derived during infection, negative selection of high affinity self-reactive TCRs in the thymus dictates that self-reactive T cells in general are dominated by T cells with low affinity TCRs for self.

To put this affinity profile in perspective, we have demonstrated that the self-reactive 2D2 TCR receptor transgenic mouse reactive for MOG_35–55_ [118]_,_ has an affinity >10,000 fold lower than the viral specific SMARTA TCR receptor transgenic mouse reactive for the GP_61–80_ epitope of LCMV. pMHC tetramer and multimers, therefore, do not readily detect antigen-reactive T cells that elicit a functional response [63,64,81,82] but despite the exceedingly low affinity of 2D2 T cells, they proliferate to MOG peptide and, crucially, they induce the symptoms of EAE [85]. The efficacy of low affinity Teff is also supported by analysis of a panel of retrogenic mice, which had T cells with TCRs of varying functional avidity for MOG_35–55_ as a surrogate readout of TCR affinity. This study concluded that T cell affinity did not determine disease pathogenesis [119]. Furthermore, in relapse/remitting EAE in non-obese diabetic (NOD) mice, the TCR affinity at the secondary progressive remitting stages was equal to or lower to other points in disease [64]. This is not to say that affinity might not alter the kinetics of disease, but rather to emphasize the point that lower affinity T cells are functional and capable of driving disease.

A second example is in T1D. A panel of eight insulin-reactive InsB_9–23_ specific retrogenic MHC II–restricted TCRs with a broad range of 2D affinity was used to probe the relationship between TCR affinity and disease [87]. Clones with intermediate affinities correlated with the earliest onset of diabetes but the lowest affinity clone induced the highest rate of diabetic incidence. The highest affinity retrogenic clone P2 and the second lowest 12–4.4m1 did not generate spontaneous disease demonstrating that higher affinity Teff are not necessarily the most functional.

### 7.2. T Follicular Helper Cells (Tfh)

Tfh are a population of T effector cells characterized by the production of IL-21 and the surface expression of PD-1 and CXCR5 driven by the transcription factor Bcl-6 [120,121,122,123]. They are critical in providing B cell help to drive humoral immune responses. Based on tetramer staining, Tfh have been suggested to be regulated by the strength of T cell antigen receptor binding [124]. We have shown that TCRs on developing Tfh have a significantly higher 2D affinity than non-Tfh effector CD4+ T cells [125]. Importantly, this differentiation corresponded to initial IL-2 production with the early high producers becoming Tfh.

Potential similarities are also seen with a population of IL-21-producing CD4+ T cells in the CNS after infection with mouse polyoma virus (MuPyV) [126]. IL-21 producers are thought to be essential for CD8+ T cell differentiation into resident memory T cells in the brain after infection with MuPyV. IL-21+ CD4+ T cells demonstrated higher 2D affinity than non-IL-21 producers [126], indicating that in this system higher affinity T cells may be protective against subsequent reactivation of MuPyV infection.

### 7.3. Tregs

For several years it has been thought that Tregs harbored TCRs with higher affinity for pMHC [127,128,129]. However, 2D-micropipette analysis of MOG reactive Tregs in EAE revealed that the affinity profile of TCRs on Teff and Tregs is in fact very similar in this model [81]. There was also a subtle, but significant, decrease in polyclonal 2D affinity for MOG_38–49_ reactive Tregs in the CNS as EAE progressed [81], suggesting a mechanism for potential escape from immune regulation as autoimmune disease advances. The idea that Tregs are not driven by high affinity interactions of TCR with pMHC is also supported by data from a mouse model of T1D where low and high affinity Tregs were recruited to the pancreas [130].

### 7.4. Memory T Cells

Memory T cells are critical to recall responses upon reinfection with the same pathogen or in the context of vaccination. One report has demonstrated that memory cells may have a different affinity profile compared to Teff [69]. Using the LCMV model of viral infection in mice, it has been shown that, at the peak of infection, Teff cells to the dominant MHC-II restricted GP_66–77_ epitope are higher affinity [69] whereas cells transitioning to memory CD4+T cells have a lower affinity. A similar observation has also been shown in MuPyV infection where memory CD8+ T cells found in the periphery (spleen) had decreased affinity as compared to the affinity at peak day 8 response [131]. The mechanisms underlying these observations are unknown but may be related to different transcriptional programs initiated by lower affinity TCR signaling programs. Memory T cells can also arise in cases where one has not seen the antigen as defined by the use of tetramers, and analysis of low and high affinity TCRs revealed similar levels of cross-reactivity [132]. The emergence of the novel severe acute respiratory syndrome coronavirus 2 (SARS-CoV-2) in late 2019 show the involvement of CD4+ T cells [133,134,135,136,137] in people who had not been exposed to SARS-CoV-2 [134,136]. Utilization of techniques to measure the range T cell affinities in such cross-reactive T cells could be critically informative for predicting patients who present with acute, chronic, and mild disease.

## 8. High and Low Affinity CD8+ T Cells

### 8.1. Effector CD8+ T Cells

Similar to CD4+ T cells, tetramer analysis can miss lower affinity CD8+ T cells and optimized tetramer staining protocols need to be employed for identification of a more complete population of antigen-reactive viral- and tumor-specific CD8+ T cells [62]. However, even with optimized approaches, tetramer staining does not include the levels of antigen-reactive T cells that are identified by 2D-micropipette. In comparison with CD4+ T cells, the idea that high affinity CD8+ T cell interactions are optimal for function during an immune response has more support. For example, data using ovalbumin (OVA) APLs as a surrogate for affinity, suggests that high affinity CD8+ T cells predominantly generate short-lived effector cells (SLEC) [75]. Furthermore, a study of human diabetogenic CD8+ T cells (HLA-A2-ALWGPDPAAA) stimulated with panel of agonist APLs to give a profile of a 3-log range of affinity demonstrated there was a strong positive correlation with affinity and the EC50 of the lytic response [138]. When these cells were stimulated, the higher affinity ligands induced more lysis than the lower affinity ligands. Importantly, all ligands induced lytic functions in this T cell line demonstrating that higher affinity interactions are not absolutely required for CD8+ T cell lytic function.

In a key cancer study of three patients who received autologous CD8+ T cells transduced with a with TIL1383I-TCR, it was found that the 2D measurements correlated with outcome. The patient with complete remission demonstrated the highest affinity and the unresponsive patient had a significantly lower affinity [139]. In another study using human tumor-reactive CD8+ T cells, lower affinity altered peptide ligands correlated with reduced calcium release indicating less activation [25]. This supports the idea that lower affinity T cells respond but at somewhat reduced levels in a cancer setting.

### 8.2. Memory CD8+ T Cells

With respect to memory cells [69], polyclonal CD8+ T cells are similar to CD4+ T cells and exhibit lower affinity during memory responses [65,76]. Again, using again the OVA based APL system with OT-I T cell transgenic cells, both high affinity (N4 pMHC-I) and low affinity (V4 pMHC-I) induced proliferation, although the lower affinity peptide induced cell division to a lesser extent [37,140]. However upon stimulation, V4 pMHC-I induced a greater differentiation of OT-I cells with a memory phenotype [76] than those cells interacting with high affinity ligands [37,140]. Given these results, one could propose that, at peak disease high affinity T cells in response to dominant epitopes would generate mostly SLEC and lower affinity T cells would favor memory precursor effector cells (MPEC). Nevertheless, not all types of memory CD8+ T cells are favored by lower affinity. Studies from two different infections in the central nervous system show that tissue resident memory CD8+ T cells (Trem) are of higher affinity. In vivo tracking of three CD8+ T cell clones reactive to epitopes of the ROP7 antigen of *Toxoplasma gondii*, found that it was the two highest affinity clones that trafficked to the brain (R7-I and R7-III) during acute infection and these clones were maintained as CD103+ Trm [141]. Similarly the CD8+ resident memory T cells in the brains of MuPyV-infected mice have also been shown to harbor higher affinity TCRs as compared to the memory cells found in the spleen [131]. Whilst more work needs to be carried out to determine if there are generalized patterns whereby memory CD8+ T cells have a predilection for a particular affinity profile, data thus far suggests that affinity may have different effects on memory T cell fate.

## 9. Conclusions

A comprehensive understanding of the varied roles of T cells during adaptive immune responses can be achieved through a focus on 2D binding kinetics of antigen recognition as it relates to effector function. The contribution of lower affinity T cells has been overlooked due to sensitivity limitations of commonly used assays often requiring multimerized pMHCs for detection. Without inclusion of all antigen-reactive T cells, which can only be identified with sensitive tools such as the 2D micropipette, interpretation of antigen specificity and T cell frequency and function can be misleading. Here, we have highlighted the importance of lower affinity T cells that have previously escaped detection in a variety of model systems. T cells with lower affinity TCRs expand and are functional with the evidence thus far suggesting there can be differences in functional phenotype. Employing sensitive measures of T cell affinity in future studies will allow for more in-depth analysis of immune responses which in turn will facilitate the development of therapeutic interventions based on information that encompasses the entire affinity range found in the T cell population.
